# The effects of trastuzumab therapy on endothelial functions of breast cancer patients

**DOI:** 10.1590/1806-9282.20240517

**Published:** 2024-09-16

**Authors:** Çağlar Alp, Mehmet Tolga Doğru, Selim Yalçın, Ali Oğuzhan Karal

**Affiliations:** 1 Kırıkkale University, Faculty of Medicine, Department of Cardiology – Kırıkkale, Turkey.; 2 Kırıkkale University, Faculty of Medicine, Department of Oncology – Kırıkkale, Turkey.

**Keywords:** Breast cancer, Trastuzumab, Cardiovascular system, Autonomic nervous system

## Abstract

**OBJECTIVE:**

Breast cancer is among the highest causes of morbidity and mortality in women. Trastuzumab therapy, which is known to be significantly cardiotoxic, is mainly used to treat patients with resistant breast cancer, including estrogen receptor-positive type. We aimed to show the effects of trastuzumab therapy on endothelial functions of breast cancer patients.

**METHODS:**

In this study, a total of 26 participants (24 female and 2 male patients, minimum age: 38 years, maximum age: 79 years, and mean age 57.3±12.7 years) were enrolled in the study. For the statistical evaluation of data, we classified the participants of the study as follows: Pretreatment: Before trastuzumab therapy; Treatment Period 1: 1 month after the first dose of trastuzumab; Treatment Period 2: 4 months after the first dose of trastuzumab; Treatment Period 3: 12 months after the first dose of trastuzumab. We conducted repeated-measures analysis of variance (Greenhouse-Geisser) and paired-sample t-tests to statistically compare the groups using flow-mediated dilation measurements.

**RESULTS:**

We determined that there are statistically significant differences between flow-mediated hyperemia and ratio values (flow-mediated dilation) of the groups (p<0.009 and p<0.001, respectively).

**CONCLUSION:**

Our data indicate that trastuzumab therapy could have negative effects on endothelial functions in breast cancer patients.

## INTRODUCTION

Considering that breast cancer is the most common type of cancer in women and causes serious clinical problems and complications, knowledge about the side effects of many forms of treatment and drugs developed for breast cancer becomes increasingly important in clinical practice^1^. Trastuzumab is a treatment agent that has achieved significant success in the treatment of breast cancer, especially in groups with positive estrogen receptors^2^.

It is a well-known fact that trastuzumab therapy has critical usefulness in the treatment of breast cancer. On the contrary, this therapy has potentially significant side effects and serious complications, especially on the cardiovascular system, which are of great clinical importance and require a more stringent monitoring of cardiovascular system complications in patients under trastuzumab treatment.

The presence of anthracyclines in the treatment protocols applied to many patients receiving trastuzumab increases the frequency and severity of possible cardiac complications. However, this sometimes causes controversy about to what extent trastuzumab and anthracyclines are responsible for such cardiac complications^2-9^.

Nevertheless, regardless of its evaluation method, the cardiotoxicity of trastuzumab is evident in many studies and is of great importance in the clinical follow-up^4,5^. Congestive heart failure (CHF) is the most common outcome observed when trastuzumab therapy is given alone or in combination with anthracyclines in particular. In view of all possible etiological factors, endothelial dysfunction constitutes one of the most important pathophysiological mechanisms underlying many pathological clinical conditions that cause CHF^10-14^, so it could be considered that endothelial dysfunction might have a critical role in the pathophysiological mechanism for trastuzumab-mediated CHF (TMCHF)^12-14^.

Flow-mediated dilation (FMD) is the most commonly used method for noninvasive assessment of endothelial functions^15-18^. Endothelial functions can be affected by a variety of chronic degenerative diseases, including atherosclerosis, diabetes mellitus (DM), and hypertension^17^. Accordingly, FMD can be useful in the evaluation of nitric oxide (NO)-releasing capacity in breast cancer patients on trastuzumab therapy^15-18^.

There is a few number of data about endothelial dysfunction associated with trastuzumab therapy in breast cancer patients in the literature. In this study, we aimed to show the negative effects of trastuzumab therapy on endothelial functions of breast cancer patients.

## METHODS

This is a cross-sectional study of 55 patients with HER-2-positive breast cancer admitted to the Oncology Department of the Medical School of Kırıkkale University, and the relevant symptoms were screened between October 2022 and October 2023. The study design was approved by the local ethics committee (ID 07/01 12.09.2022).

After the objectives of the study were described to the patients and their written informed consents were obtained, baseline characteristics and clinical data of the participants were collected by an interview and recorded in the study questionnaire and data from FMD analysis.

### Patients

All participants were evaluated in Oncology and Cardiology clinics. Cardiologic evaluation and measurements, including FMD measurements, were performed by a cardiologist.

### Patient selection

Exclusion criteria were stable or unstable angina pectoris, acute myocardial infarction, systolic heart failure (ejection fraction (EF)<50%), hypertension, valvular heart disease, aortic aneurysm, acute or chronic renal failure (serum creatinine level >1.5 mg/dL), DM, asthma or chronic obstructive lung disease, neurological and psychiatric diseases, and alcohol and drug abusement.

A total of 29 patients were excluded because of developing a condition included in the exclusion criteria during the study period.

A total of 26 participants (24 female and 2 male patients, minimum age: 38 years, maximum age: 79 years, and mean age: 57.3±12.7 years) were enrolled in the study.

We classified the study participants as follows:

- Group 1: Pretreatment: Before trastuzumab therapy
- Group 2: (Treatment Period 1) 1 month after the first dose of trastuzumab
- Group 3: (Treatment Period 2) 4 months after the first dose of trastuzumab
- Group 4: (Treatment Period 3) 12 months after the first dose of trastuzumab.

### Oncologic evaluation

A total of 26 patients evaluated for the study were early-stage HER-2-positive [3 positive by immunohistochemistry or positive by fluorescence in situ hybridization (FISH) technique] and completed their local treatment (modified radical mastectomy and axillary dissection) with at least four courses of adjuvant anthracycline-based chemotherapy followed by trastuzumab (the first dose 8 mg/kg, after that, maintenance dose 6 mg/kg) every 3 weeks) intravenous treatment for 1 year was planned.

All patients had completed adjuvant radiotherapy, during which they received 2 Gy/fraction/day, a total of 40–60 Gy (mean 52 Gy) of radiotherapy.

### Cardiologic evaluation

Following a detailed medical history taking, all subjects were physically examined and their blood pressures were measured in both arms using a sphygmomanometer. 12-channel electrocardiography (ECG) recordings and transthoracic echocardiography (Ge-Vivid 7 Pro, General Electric; FL, USA,), FMD, and PWA tests were performed.

#### Flow-mediated dilation

A Ge-Vivid 7 Pro, 12 L Doppler probe (General Electric, Florida, USA) was used to detect the measurements of flow-mediated dilation. Flow-mediated dilation measurements were performed as defined by Hayward et al^18^.

In the present study,

- basal brachial artery diameter measurements were represented as FMD basal (cm),
- the brachial artery diameter at Hyperemia phase was represented as FMD hyperemia (cm), and
- we also calculated the FMD basal/FMD hyperemia ratio (%)^15,17^.

### Statistical analysis

All statistical analyses were conducted using SPSS version 20.0 (SPSS; Chicago, IL, USA). According to statistical distribution types, we showed the data as mean±standard deviation (SD) which have normal distribution. Besides, we showed the data as median (25–75%) which have non-normal distribution. Repeated-measures analysis of variance (ANOVA) (Greenhouse-Geisser) and paired sample t-tests were employed to compare data of the groups. A p-value of <0.05 was accepted as statistically significant.

## RESULTS

Table 1 shows FMD measures of all participants of the study.

**Table 1:** Statistical comparison of flow-mediated dilation measurements of breast cancer patients under trastuzumab therapy.

Patient characteristics	Pretreatment	Treatment Period 1	Treatment Period 2	Treatment Period 3	p
FMD basal (mm)	3.88±0.61	3.82±0.56	3.83±0.53	3.90±0.48	0.139
FMD hyperemia (mm)	4.21±0.64	4.08±0.57	4.03±0.54	4.04±0.48	**0.009**
FMD ratio (%)*	92.10±3.65	93.51±3.34	95.07±3.60	96.60±2.38	**<0.001**

Bold values are statistically significant measures.

Repeated-measures ANOVA (Greenhouse-Geisser) test, Mean±SD, p<0.05.

* FMD Ratio: [FMD basal/FMD hyperemia]x100. Pretreatment: Before trastuzumab therapy. Treatment Period 1: 1 month after the first dose of trastuzumab. Treatment Period 2: 4 months after the first dose of trastuzumab. Treatment Period 3: 12 months after the first dose of trastuzumab.

There was no statistically significant difference between the FMD basal diameters of the groups. However, there were statistically significant differences in FMD hyperemia values between the groups (p<0.009) (Table 1). After the Bonferroni adjustment test, we found a significant difference in FMD hyperemia between Group 1 and Group 3 (p=0.027).

**Figure 1:**
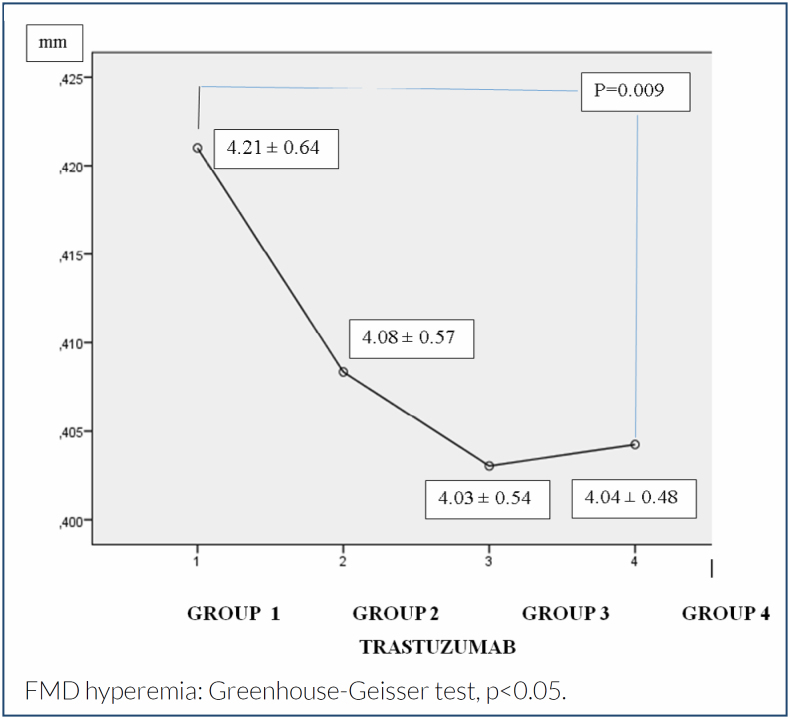
Variation of flow-mediated diameter hyperemia values with time periods in breast cancer patients treated with trastuzumab.

**Figure 2:**
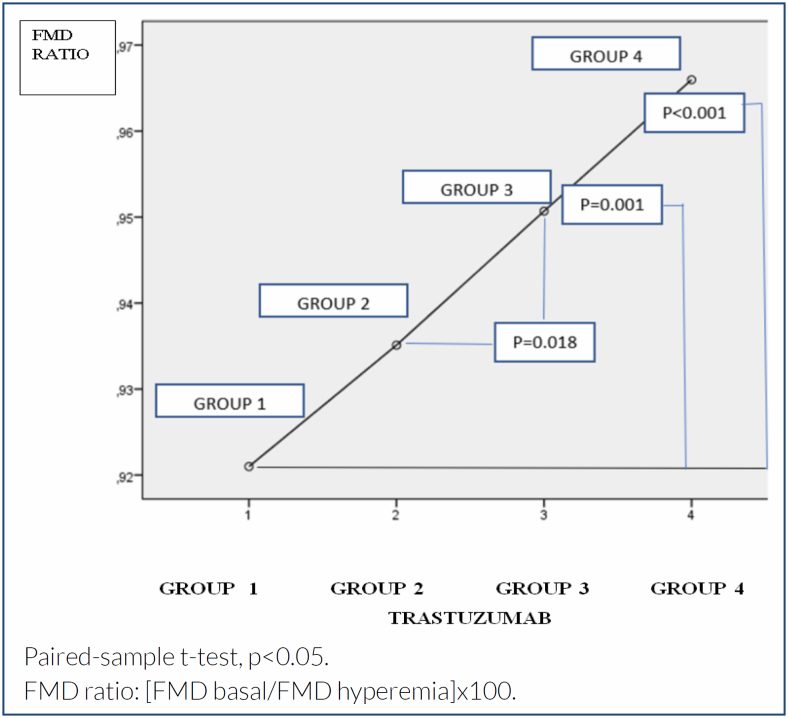
Variation of flow-mediated diameter ratio values with time periods in breast cancer patients treated with trastuzumab.

Our results have shown that there was a significant decrease in the hyperemia capability of the endothelial layer according to trastuzumab therapy (Figure 1). As FMD ratio values are derivatives of FMD basal and hyperemia, a similar change is seen in FMD ratio values. According to our results, there are significant decreases in FMD ratios during treatment (Table 1). Besides, we identified significant differences in FMD ratios between Group 1 and Group 3 (p=0.001), Group 1 and Group 4 (p<0.001), and Group 2 and Group 3 (p=0.018) (Figure 2).

## DISCUSSION

In this study, decreased FMD hyperemia and increased FMD ratio (basal/hyperemia diameters) values were found during trastuzumab therapy. As the FMD test results showed in our study, progressively increasing endothelial dysfunction is observed in four consecutive evaluations during the 1-year treatment period.

Endothelial cells, which constitute one of the most important cell groups of the entire cardiovascular system, both structurally and functionally, have an indispensable importance in the holistic functioning of the cardiovascular system^19^. Many pathological conditions affecting endothelial cells are known to constitute the basic pathophysiological mechanisms of cardiovascular diseases. However, the factors that negatively affect endothelial cells structurally and functionally also include drugs used to treat various diseases.

The most important of these drug groups are different types of treatment agents used in cancer chemotherapy. The mechanisms by which cancer chemotherapy causes toxic effects on endothelial cells are not fully known. It is common knowledge that the main function of endothelial cells is to dynamically maintain vascular permeability and tone under different biological requirements. Many different mediators are involved in the fulfillment of this function at the tissue and cell level. Among these factors, vascular endothelial growth factor (VEGF) that mobilizes endothelial progenitor cells for vascular repair, IL-6, TNFα, and intercellular adhesion molecule 1, NO, reactive oxygen species (ROS), and platelet activation are the most important ones^20^.

Trastuzumab is one of the most important chemotherapeutic drugs that have structural and functional toxic effects on endothelial cells used in cancer chemotherapy^21^. Many studies have found that cardiotoxicity related to trastuzumab is closely related to age, obesity, hypertension, coronary artery disease, and concomitant anthracycline therapy^6-9^. These factors are also known as endothelial dysfunction.

The toxic effect of trastuzumab, acting by blocking the HER2 signaling pathway, which is of great importance in putting the structurally and functionally normal functions of the cardiovascular system on the route, can also be observed clinically in many patients^10^. The HER2 signaling mechanism is related to NO generation in the endothelial layer^11^.

It has been demonstrated in many different animal experiments that pathological changes in the HER2 signaling pathway cause clinical conditions such as cardiomyopathies and related heart failure^22^. The HER2–HER4 heterodimerization mechanism actually constitutes a protective mechanism against the effects of many external toxic agents in endothelial and cardiac muscle cells. On the contrary, trastuzumab blocks this mechanism and exposes the endothelium, including itself, to the effects of many toxic agents^23^. In many different conditions, the amount of ROS increases in endothelial dysfunction. Meanwhile, angiotensin II (ANG II), thrombin, and nicotinamide adenine dinucleotide phosphate-oxidase (NADPH) with a negative positive feedback mechanism are the most important factors that increase ROS^24^. There are a lot of studies that have shown that the expression of the endothelial nitric oxide synthase (eNOS) gene is important for endothelial functions and simultaneously survival following trastuzumab treatment. Besides, many of the studies have also shown that eNOS gene polymorphisms could cause negative effects on survival in breast cancer patients and the patients with higher expression of eNOS in the microvessels have better prognoses too^25^.

It seems that the alteration of NO production capacity during trastuzumab therapy could be a representative of trastuzumab toxicity. The findings of our study are consistent with previous studies. Evaluations at the end of different periods of trastuzumab therapy indicated progressive endothelial dysfunction, as demonstrated by FMD tests in our study.

## CONCLUSION

Our data showed that the patients receiving trastuzumab therapy had deteriorated flow-mediated dilation responses, suggesting endothelial dysfunction caused by this treatment.

## STATEMENT OF ETHICS

The study protocol was approved by the Kirikkale Üniversity Ethics Committee, Kirikkale, Turkey (Date/Number: 12.06.2022 07/01).

## Data Availability

All data relevant to this study will be provided by the authors upon specific request.
